# Validation of a blood protein signature for non-small cell lung cancer

**DOI:** 10.1186/1559-0275-11-32

**Published:** 2014-08-01

**Authors:** Michael R Mehan, Stephen A Williams, Jill M Siegfried, William L Bigbee, Joel L Weissfeld, David O Wilson, Harvey I Pass, William N Rom, Thomas Muley, Michael Meister, Wilbur Franklin, York E Miller, Edward N Brody, Rachel M Ostroff

**Affiliations:** 1 SomaLogic, Inc., Boulder, CO, USA; 2 University of Pittsburgh Cancer Institute, School of Medicine, Pittsburgh, PA, USA; 3 University of Minnesota, Minneapolis, MN, USA; 4 Department of Epidemiology, Graduate School of Public Health, University of Pittsburgh, Pittsburgh, USA; 5 Langone Medical Center and Cancer Center, New York University School of Medicine, New York, NY, USA; 6 New York University School of Medicine, New York, NY, USA; 7 Thoraxklinik at University Hospital Heidelberg and Translational Lung Research Center Heidelberg (TLRC-H), Member of the German Center for Lung Research (DZL), Heidelberg, Germany; 8 University of Colorado Cancer Center, University of Colorado School of Medicine, Aurora, CO, USA; 9 Denver Veterans Affairs Medical Center, Denver, CO, USA

**Keywords:** Lung cancer, Biomarker, SOMAmer, Proteomic, Squamous cell carcinoma, Diagnosis, Preanalytic variability, Sample bias

## Abstract

**Background:**

CT screening for lung cancer is effective in reducing mortality, but there are areas of concern, including a positive predictive value of 4% and development of interval cancers. A blood test that could manage these limitations would be useful, but development of such tests has been impaired by variations in blood collection that may lead to poor reproducibility across populations.

**Results:**

Blood-based proteomic profiles were generated with SOMAscan technology, which measured 1033 proteins. First, preanalytic variability was evaluated with Sample Mapping Vectors (SMV), which are panels of proteins that detect confounders in protein levels related to sample collection. A subset of well collected serum samples not influenced by preanalytic variability was selected for discovery of lung cancer biomarkers. The impact of sample collection variation on these candidate markers was tested in the subset of samples with higher SMV scores so that the most robust markers could be used to create disease classifiers. The discovery sample set (n = 363) was from a multi-center study of 94 non-small cell lung cancer (NSCLC) cases and 269 long-term smokers and benign pulmonary nodule controls. The analysis resulted in a 7-marker panel with an AUC of 0.85 for all cases (68% adenocarcinoma, 32% squamous) and an AUC of 0.93 for squamous cell carcinoma in particular. This panel was validated by making blinded predictions in two independent cohorts (n = 138 in the first validation and n = 135 in the second). The model was recalibrated for a panel format prior to unblinding the second cohort. The AUCs overall were 0.81 and 0.77, and for squamous cell tumors alone were 0.89 and 0.87. The estimated negative predictive value for a 15% disease prevalence was 93% overall and 99% for squamous lung tumors. The proteins in the classifier function in destruction of the extracellular matrix, metabolic homeostasis and inflammation.

**Conclusions:**

Selecting biomarkers resistant to sample processing variation led to robust lung cancer biomarkers that performed consistently in independent validations. They form a sensitive signature for detection of lung cancer, especially squamous cell histology. This non-invasive test could be used to improve the positive predictive value of CT screening, with the potential to avoid invasive evaluation of nonmalignant pulmonary nodules.

## Background

Lung cancer is the 5^th^ leading cause of death worldwide. There were 1.5 million lung cancer deaths in 2010, an increase of 48% in the last 20 years [1]. Although lung cancer death rates are declining in developed countries, lung cancer incidence and death rates are rapidly rising in the developing world, where smoking prevalence continues to increase [2]. Most patients present with advanced disease and 5-year survival rates are poor, ranging from less than 10% in China to 13-16% in Europe and the US [3-5].

The National Lung Screening Trial (NLST) demonstrated that screening smokers and ex-smokers for lung cancer can lead to early diagnosis and reduced lung cancer mortality [6]. However, the low (4%) positive predictive value (PPV) of CT screening in the NLST cohort leads to a large number of unnecessary follow-up procedures, including surgery for benign nodules, as was first reported in the Pittsburgh Lung Screening Study (PLuSS) and later in the NLST [6,7]. The European NELSON CT screening study includes tumor volume and doubling time in the assessment of pulmonary nodules and improves the PPV to 41% by only referring small nodules (50-500 mm^3^) for clinical follow-up if they show evidence of growth and a doubling time of less than 400 days [8,9].

Squamous cell carcinoma (SQ) accounts for approximately 30% of non-small cell lung cancer (NSCLC) cases and is closely associated with long term tobacco exposure while adenocarcinoma (AD) is the most common form of NSCLC, even more so in never or light smokers [10]. AD tumors often present in the periphery and are the most prevalent histology detected by CT [11-13]. On the other hand, SQ tumors typically arise in the central airway and frequently are in close proximity to main blood vessels [14], which may contribute to better detection of tumor biomarkers in the blood. SQ are the most common NSCLC histology missed by CT [6,15], perhaps because a central tumor location obscures CT detection and because SQ tumors tend to have a rapid growth rate that can lead to diagnosis as interval cancers between scans. In a Taiwan population-based registry of over 33,000 lung cancer patients, those with SQ tumors had shorter median survival times than adenocarcinoma (AD) tumors [16].

Detterbeck and Gibson’s review of the natural history of lung cancer reports that conventionally detected NSCLCs have rapid doubling times averaging 135 days, while those detected in CT screening programs have substantially slower growth rates of 480 days [15]. They also report that the average doubling time of AD is 576 days and SQ is 122 days, which is consistent with twice as many SQ than AD detected as late stage disease in CT screening [12]. The AD average doubling time is longer than the cutoff for a positive screening result established by the NELSON trial of 400 days [9]. Observations such as these have led to speculation that while CT screening clearly leads to a mortality benefit for lung cancer, the risk of over-diagnosis must be considered [17,18]. In particular, 22% of NSCLC and 79% of bronchioalveolar lung cancers detected by CT were reported as indolent cancers and possible cases of overdiagnosis [19]. Overdiagnosis of indolent cancers may lead to increased costs, anxiety and harms from unnecessary invasive procedures.

We previously reported the discovery of a protein biomarker panel for early detection of lung cancer [20]. We also noted that several potential serum markers were both lung cancer markers and markers of preanalytic variability. Preanalytic bias in biomarker discovery studies is a well-known culprit contributing to failed validation studies [21]. This preanalytic variability can arise from differences in blood sample collection, processing and/or storage between study sites, or worse, introduce case/control bias in samples collected differently at the same study site, mimicking disease biomarkers. Such markers will not correctly classify cases and controls in independent validation studies nor will they produce robust clinical assays.

To better understand the effect of different blood sample processing procedures, we evaluated protein measurement bias in several clinical collections and controlled laboratory studies [22-24]. These analyses revealed that perturbations in serum processing protocols result in changes to many proteins in a coordinated fashion. We subsequently developed protein biomarker signatures of processes such as cell lysis, platelet activation and complement activation and assembled these preanalytic signatures into quantitative multi-dimensional Sample Mapping Vector (SMV) scores [22]. The SMV score provides critical evaluation of the quality of every blood sample used in discovery, and also enables the evaluation of candidate protein biomarkers for resistance to preanalytic variability.

Although the AUC of 0.9 in our original report for both training and blinded verification was promising, we had to eliminate some markers, such as HSP90, due to preanalytic bias leading to site-to-site differences. When the SMV scores of preanalytic effects were applied retrospectively, we found there were substantial center-specific differences in preanalytic variation between blood samples of cases and controls, leading to artificial biomarker associations with lung cancer. As a result, our initial diagnostic performance was partly dependent on markers that not only related to cancer biology, but that were also confounded by preanalytic case/control bias.

To eliminate this effect, we used the SMV score to define a fraction of the original sample set with minimal differences in preanalytic variability between cases and controls for biomarker discovery and classifier training. The underlying platform technology used SOMAmers (Slow Off-rate Modified Aptamers) as affinity reagents to quantify 1033 proteins simultaneously with sub-pM limits of detection and inter-assay CV of <5% [25,26]. The 7-marker classifier reported here performed consistently through training and two independent blinded validation studies, illustrating that robust biomarker discovery is enabled by both careful construction of clinical cohorts and avoidance of preanalytic bias.

## Results

### Training a robust lung cancer classifier

**Figure 1:**
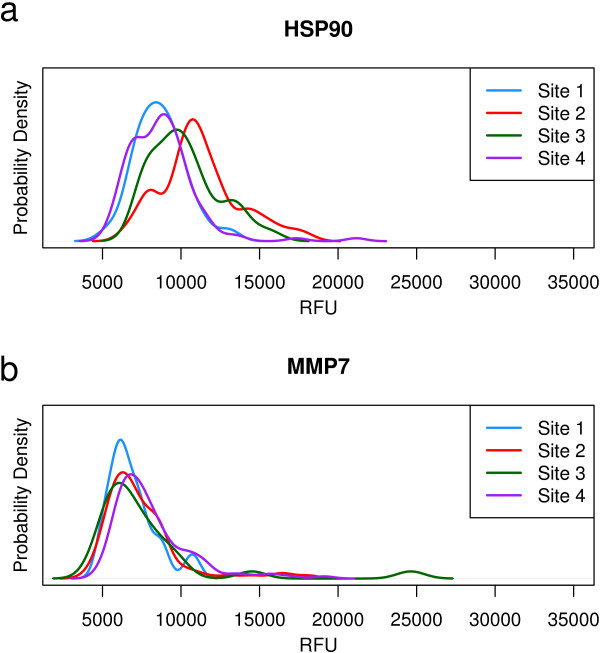
**Probability Density Function plots of HSP90 and MMP7 distributions for each training site control group. (a)** HSP90 is an example of a protein affected by preanalytic variability and the plot demonstrates the bias between control groups. **(b)** MMP7, selected from the high quality training samples, is consistent between sites.

In our initial lung cancer study we identified numerous NSCLC biomarkers [20]. We also reported that this analysis was confounded by large scale differences between the study sites. This observation motivated a series of studies investigating the effect of sample handling on biomarker discovery [22,23]. The result of these studies was the development of a set of SMVs which allowed us to quantify the magnitude of confounding pre-analytical variation introduced by sample collection differences.For example, the cytoplasmic molecular chaperone HSP90 is a well established lung cancer marker and therapeutic target. We measured elevated levels of HSP90 in the serum of lung cancer cases and included it in our original classifier. However, HSP90 levels in serum are affected by cell lysis during sample processing and intracellular protein contamination leaking into serum, causing a relative shift in the measured concentration. We compared the distribution of HSP90 in the control group by study sites (Figure 1a) and found that the mean level varied by more than 2-fold across study sites. We concluded that even though HSP90 is a strong lung cancer marker, serum protein levels are confounded by preanalytic variability and thus not robust enough for routine clinical testing. These observations led us to embark on a new effort to discover robust lung cancer biomarkers.Using the cell contamination and complement SMVs, we selected a subset of the original samples that were uniformly collected. We also included a small set of more variable samples to test the consistency of our biomarkers. Samples were categorized as uniformly collected or variable based on empirically derived SMV cutoffs, as described in the Methods section. This strategy allowed us to discover robust biomarkers that were not affected by sample handling. MMP7 is one of the markers identified by this strategy. In contrast to HSP90, the distribution of MMP7 across control groups is consistent, demonstrating that this strategy yielded biomarkers immune to common preanalytic variability seen across different serum collections (contrast Figure 1a and b).

The training set consisted of 363 serum samples from 4 clinical sites (Table 1). The control population represents a high risk population for lung cancer and included long term smokers, 45% with benign pulmonary nodules. The intensity and duration of tobacco exposure was balanced between the cases and controls. The cases were 68% AD and 32% SQ, consistent with the current US diagnosis pattern and 53% were localized, resectable tumors (stages I and II).

**Table 1:** Demographics of training and validation study cohorts

**Characteristic**	**Training (n = 363)**		**UHH validation (n = 138)**		**EDRN validation (n = 135)**	
	**Case**	**Control**	**Case**	**Control**	**Case**	**Control**
No. subjects	94	269	111	27	63	72
Median age (years)	69	57	62	58	68	71
Interquartile range	63-74	52-64	54-70	51-71	62-74	66-76
Gender						
Male	42	126	70	13	36	39
Female	52	143	41	14	27	33
Median pack-years*	40	40	35	30	60	38
Interquartile range	23-57	20-56	20-50	18-43	40-76	34-60
Histopathology/Stage						
Adenocarcinoma	64		55		38	
I	27		19		24	
II	7		16		5	
III	19		20		7	
IV	11		0		2	
Squamous cell	30		56		25	
I	11		20		14	
II	5		16		4	
III	12		20		3	
IV	2		0		4	
Benign nodule		122		27		20

*Pack-years is defined as the product of the total number of years of smoking and the average number of packs of cigarettes smoked daily.

Smoking data was not available for 8 subjects in training, 21 in UHH and 10 in EDRN studies.

**Figure 2:**
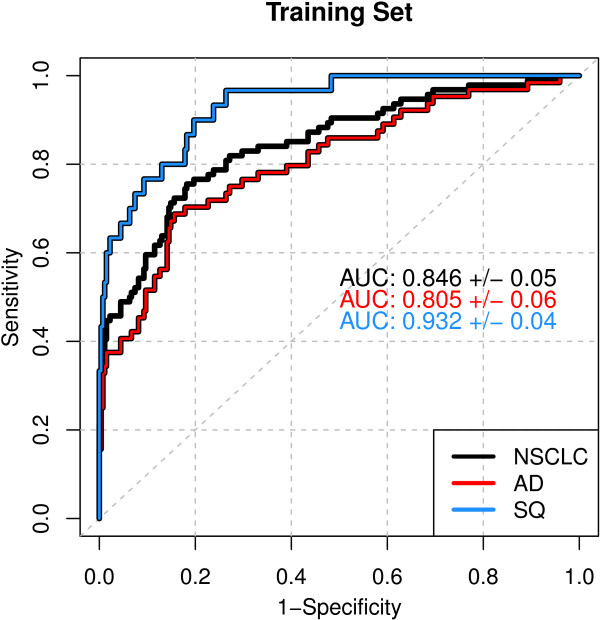
**Training set ROC.** Results are plotted for the entire data set and for AD and SQ tumor histologies separately.

We generated proteomic data with SOMAscan V2, which measures 1033 analytes, an increase of 232 proteins over V1 used in the original lung cancer studies [20]. From this new analysis, we identified 15 candidate biomarkers (Additional file 1) and built a 7-protein Random Forest (RF) model with an AUC of 0.85 for all training samples (Figure 2 and Table 2). Although 68% of the cases were AD, the classifier was better at detecting SQ with an AUC of 0.93 (Figure 2). The differential expression of all of the classifier proteins was also greater for SQ than AD compared to controls. Even though the majority of cases were AD, models built from the training data consistently performed better with SQ. This led us to design a study to examine the histological sensitivity.

**Table 2:** Lung cancer classifier proteins ranked by gini importance score

**Protein [Entrez gene ID]**	**Name**	**Gini importance**	**Function**	**Up/down in NSCLC**
MMP12 [4321]	Matrix metallo-peptidase 12	20.33	Breakdown of extracellular matrix, positive regulation of cell proliferation, tissue injury and remodeling	Up
SERPINA3 [12]	Apha-1 antiproteinase	14.11	Serine protease inhibitor, part of acute phase response and tissue homeostasis	Up
MMP7 [4316]	Matrix metallo-peptidase 7	13.73	Breakdown of extracellular matrix, positive regulation of cell proliferation, collagen catabolism, degrades fibronectin	Up
C9 [735]	Complement component 9	11.31	Inflammatory acute phase reactant, pore-forming subunit of cytolytic MAC complex	Up
CRP [1401]	C-reactive protein	11.01	Inflammatory acute phase reactant, immune effector	Up
CNDP1 [84735]	Carnosine dipeptidase 1	8.66	Carboxypeptidase, functions in amino acid transport and metabolism	Down
CA6 [765]	Carbonic anhydrase VI	7.62	Reversible hydratation of carbon dioxide, one-carbon metabolism, nitrogen metabolism	Down

### Validation studies and histological performance

**Figure 3:**
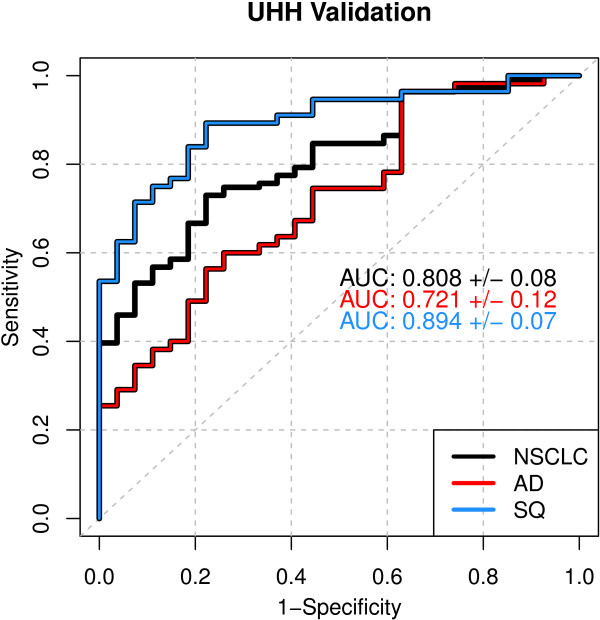
**UHH validation ROC.** Results are plotted for the entire data set and for AD and SQ tumor histologies separately.

**Figure 4:**
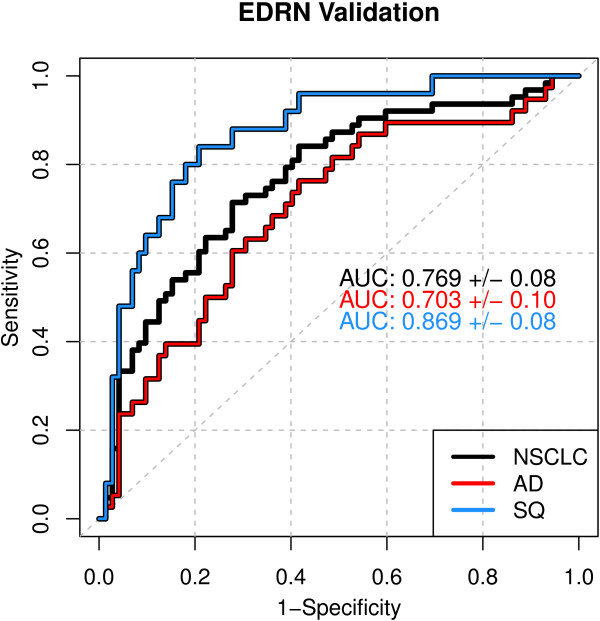
**EDRN validation ROC.** Results are plotted for the entire data set and for AD and SQ tumor histologies separately.

To precisely determine the histological sensitivity of the classifier, we designed a nested case/control study from samples obtained from the University Hospital Heidelberg (UHH) that balanced stage and AD/SQ histology. A small number of benign nodule controls were included to verify specificity. This independent, blinded study confirmed the performance established in training with an AUC of 0.81. The AUC increased to 0.89 when only the SQ cases were considered (Figure 3).A second independent, blinded validation study was performed with a reference set from the National Cancer Institute (NCI) Early Detection Research Network (EDRN) designed to validate candidate lung cancer markers for diagnosis of pulmonary nodules. The 63 cases were 46% Stage I/II AD and 29% Stage I/II SQ. Late stage cases made up 25% of the cohort and were approximately equally distributed between AD and SQ. The controls were smokers who had undergone radiologic testing for suspicion of lung cancer and had received a non-malignant diagnosis. The AUC for all samples in this study was 0.77, which is statistically equivalent to the training set (Figure 4). Only 33% of these samples would fall into the “good” processing quality category based on SMV analysis, yet the results were equivalent to the training set, supporting our hypothesis that selecting markers robust to sample variability would result in a validated classifier. Dividing the cases by histology, the AUC for SQ was 0.87 and 0.70 for AD.

The goal of lung cancer early detection is to identify the malignancy at the stage where surgical cure is possible. To illustrate the utility of our test for this clinical application, we divided test performance by stage and histology for each clinical cohort (Table 3). Sensitivity for detection of stage I AD ranged from 33-50% and stage I SQ was 64-93%. Sensitivity increased with lung cancer stage, illustrating that the markers correlate with disease burden. Sensitivity for detection of stages II-IV ranged from 51-79% for AD and 82-84% for SQ. Assuming a 15% prevalence rate based on NLST data for 11-30 mm pulmonary nodules [27], the estimated positive predictive value (PPV) for the EDRN validation set is 30% and the negative predictive value (NPV) is 93%. Considering each histology separately and halving the prevalence to 7.5%, the SQ PPV is 20% and the NPV is 99%. The PPV/NPV for AD at the same prevalence is 15%/96%. The higher estimated PPV and NPV for SQ may relate to the faster doubling time of this aggressive tumor leading to release of greater quantities of biomarker proteins into the blood.

**Table 3:** Performance of the classifier in training and validation studies

	**Sensitivity (%)**				**Specificity (%)**	
	**Stage I**		**Stage II-IV**		**Controls**	
	**AD**	**SQ**	**AD**	**SQ**	**Benign nodule**	**All controls**
Data set						
Training set	33	64	59	84	89	90
95% CI	19-52	35-85	43-74	62-95	82-93	86-93
UHH validation	35	75	51	83	81	81
95% CI	18-57	53-89	36-67	68-93	63-92	63-92
EDRN validation	50	93	79	82	70	71
95% CI	31-69	66-100	52-93	51-96	48-86	59-80

Sensitivity of the classifier in training and validation is calculated by tumor stage and histology. Specificity is calculated for the benign nodule subset and for all controls. 95% CI is the 95% confidence interval.

The proteins in the classifier function in destruction of the extracellular matrix, metabolic homeostasis and inflammation. MMP7 and MMP12 levels are elevated in lung cancer, which may facilitate breakdown of the extracellular matrix and tumor spread. The two metabolic enzymes in the classifier, CNDP1 and CA6, are lower in the serum of lung cancer patients, perhaps in response to the low pH created by increased tumor glycolysis. The proteins related to host defense, CRP, C9 and SERPINA3, are elevated in lung cancer and may arise from tumor-induced stromal inflammation. The functions represented by these biomarkers are predominant hallmarks of neoplastic growth and malignant spread. When measured together they form a sensitive signature for detection of NSCLC.

## Discussion

The successful validation of biomarker studies depends on two key aspects of biomarker discovery: identifying biomarkers in clinically relevant cohorts and controlling preanalytic sample variability that may introduce bias in the apparent disease biomarkers. We have described a series of studies for discovery and validation of a NSCLC classifier that mirrors the intended clinical use of these markers for the diagnosis of lung cancer in a high risk population.

Realizing that some of the serum lung cancer biomarkers in our previously reported classifier [20] were influenced by bias that was related to blood processing differences, such as contamination by intracellular proteins caused by cell lysis during processing, we set out to identify a new set of unbiased markers. Applying quantitative SMV measures of preanalytic variability, we identified preanalytic sample variation that revealed unintentional differences inherent in how biological samples are obtained, processed and stored. We discovered 15 NSCLC biomarkers with a well-collected sample set from 4 independent study sites that continued to perform across a wide spectrum of sample handling parameters. Nine of these were also identified in our original study [20]. From these 15 biomarkers, a robust 7-marker random forest (RF) classifier was developed with an AUC of 0.85 in training. The reliability and consistency of this classifier was demonstrated in two independent blinded validation studies with an AUC of 0.81 in the histologically balanced UHH study and 0.77 in the EDRN indeterminate pulmonary nodule cohort. The classifier sensitivity correlates with cancer stage, meaning that the biomarker levels are proportional to the extent of disease burden. We have observed that biomarkers that do not correlate with disease burden may be influenced by preanalytic bias or demographic case/control differences.

At an estimated prevalence of 15%, our PPV/NPV in the EDRN validation study was 30%/93% over all histologies. This is an improvement over the 85% NPV if all nodules were considered benign at this prevalence. To estimate NPV separately for each histology, we reduced the prevalence to 7.5% and calculated an NPV of 96% for AD and 99% for SQ, suggesting the classifier will aid in discriminating the large number of CT-identified benign nodules from true NSCLC. The consistent performance of our classifier established in training and maintained in two blinded validation studies with samples from independent study sites demonstrates the strength of our biomarker identification strategy. Choosing reliable biomarkers resistant to preanalytic variation led to a robust classifier. Consistency from training to validation has met with difficulty in other recent lung cancer biomarker reports, where a significant drop in the AUC from training to validation was reported [28,29].

We observed that quantitative measurement of well established lung cancer markers such as HSP90 can be influenced by variation in sample handling that causes an imbalance in distribution between cases and controls across study sites. This observation is similar to changes in cytokine measurements reported by Pine *et al.*[30]. These authors describe different cytokine concentration ranges in two large lung cancer cohorts, NCI-MD and the Prostate Lung Colorectal and Ovarian Cancer Screening Trial (PLCO), making it difficult to use the same case/control cutoffs for both study populations. They postulate that differences in sample handling may contribute to this bias and suggest that follow-up studies investigate standardization methods for measuring these markers. We have opted for a different solution – select robust markers that are resistant to measurement drift caused by preanalytic sample processing differences. While this approach may sacrifice some performance, the gain in reliable measurements is critical for ultimate success in the clinical setting.

The proteins in our classifier are related to the biology of tumor growth and function to sustain proliferation and activate invasion (MMP7 and MMP12), and respond to oxidative stress and deregulation of cellular energetics (CNDP1 and CA6). The supportive role of the tumor micro-environment is represented by proteins involved in avoiding immune destruction and inducing tumor-promoting inflammation (CRP, C9, SERPINA3). Extensive genomic characterization of SQ lung cancers by The Cancer Genome Atlas Research Network (TCGA) revealed dysfunctions in genes involved in these processes, including the oxidative stress response, cell cycle control, apoptotic signaling and avoiding immune detection and destruction [31].

The matrix metalloproteinases are critically important for extracellular matrix remodeling. Proteolytic matrix degradation by the MMPs promotes tumor growth, invasion and angiogenesis [32]. Overexpression of MMP7 correlates with poor NSCLC prognosis [32,33]. MMP12 expression may be induced by smoking and trigger inflammation, leading to emphysema and lung cancer in mouse models of inflammatory triggers of oncogenesis [34].

The two metabolic enzymes, CNDP1 and CA6, are both down-regulated in our NSCLC studies. CNDP1 cleaves carnosine. Lower CNDP1 activity may lead to higher levels of carnosine, which is a scavenger of reactive oxygen species (ROS) and nitrogen species [35]. Carnosine can also act as a hydrogen ion buffer. CA6 is the secreted isoform of carbonic anhydrase, and it is involved in pH, respiration and CO_2_ homeostasis [36]. The catalytic activity of each of these enzymes releases a proton and lowers the local pH. Tumors are often hypoxic and have an acidic pH as a byproduct of increased glycolysis (the Warburg effect [37]). We speculate that lowering the activity of both of these enzymes could counteract the low pH produced by increased glycolysis in tumors.

Smoking causes chronic inflammation and release of ROS, which plays an important role in tumorigenesis. Increased macrophage and neutrophil infiltration results in an increase in cytokines, growth factors and mediators of inflammation that can induce epithelial-mesenchymal transition and destruction of host cell-mediated immune responses, leading to lung carcinogenesis [38]. Three of the classifier markers, CRP, C9 and SERPINA3, function in the acute phase host response and inflammation. CRP has long been associated with lung cancer risk and prognosis [30,39,40]. Recently Shiels and colleagues reported that elevated circulating inflammation markers are associated with lung cancer risk, with CRP having the highest risk related odds ratio [41].

The complement cascade may contribute to tumor growth by promoting acute and chronic inflammation and by facilitating cellular proliferation and invasion [42]. The membrane attack complex (MAC) is the terminal event of the complement cascade, forming a pore in the cell membrane leading to target cell death. The MAC is composed of C5b, C6, C7, C8 and multiple C9 molecules, and has been shown to promote extracellular matrix disintegration, leading to invasion and metastasis [42].

Lastly, SERPINA3 is a member of the alpha-1 antiproteinase family and member of the serine protease inhibitor class. Other members of this family have also been reported as serum biomarkers for lung cancer [39,43]. These proteins function in the host defense against the tumor and increasing levels in the blood arise from migration of inflammatory cells into the tumor and systemic innate and adaptive responses.

A limitation of this study is that the biomarkers were selected to perform well across both AD and SQ tumors and that detection of other histological classes was not assessed. In light of the emerging genetic profiles from the TCGA Network and driver mutation divergence based on histology, training for AD and SQ separately could reveal histology-specific biomarkers and improve early stage detection for both tumor types. A potential limitation of incorporating host response markers into the classifier is lack of specificity. However, by incorporating multiple proteins into a classifier, rather than relying on a single biomarker, the resultant algorithm can add disease specificity. Another emerging strategy for improving biomarker panel accuracy is to incorporate biomarker measurements with clinical risk factors, particularly in the management of indeterminate pulmonary nodules [44-46].

## Conclusions

A major strength of our classifier is the sensitive detection of early stage tumors, and in particular Stage I SQ. Typically, SQ grows rapidly and is most often diagnosed at a late stage. Both the fast doubling time and central location complicate CT detection of SQ lung cancers. The validated test described here could complement CT by identifying individuals at the highest risk and improve early detection of these aggressive lung tumors. Optimizing identification of rapidly growing tumors may also reduce over-diagnosis of indolent disease.

This study highlights the importance of sample quality assessment using a tool for evaluating bias in case/control studies before proceeding to biomarker discovery. Choosing biomarkers with not just the best case/control discrimination but that are also resistant to sample processing variation increases the likelihood that a robust biomarker panel will consistently perform well in the intended clinical setting.

## Methods

### Objectives

The objective of this study was to apply the SOMAscan proteomic assay to discover and validate serum biomarkers for detection of NSCLC in current and former smokers who are at high risk for lung cancer, and to perform subset analysis based on histopathologic classification.

### Participants

**Figure 5:**
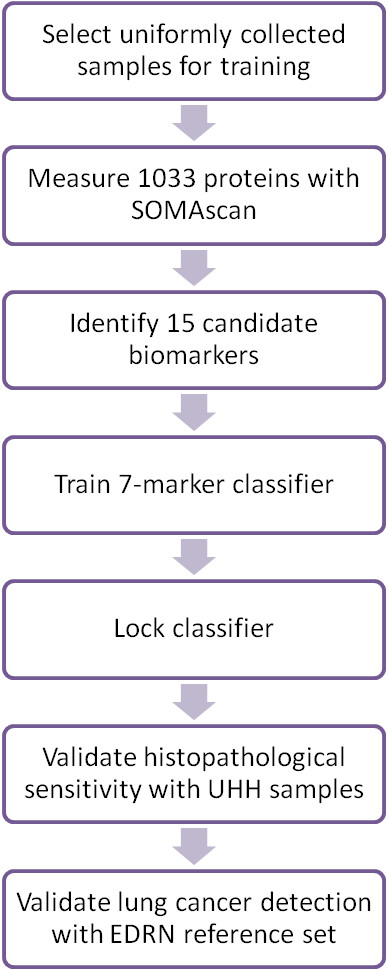
Study flowchart for biomarker discovery and validation studies.

Three prospectively designed case/control studies were performed from archived samples (Table 1). A multi-center study was conducted for biomarker selection and classifier training followed by two independent blinded validation studies from archived collections assembled by the Thoraxklinik at UHH and the EDRN (Figure 5). Inclusion criteria for cases in all three studies were: (1) diagnosis of primary lung cancer pathologically confirmed as either AD or SQ (2) no prior history of lung or other cancer except non-melanoma skin cancer in the last 5 years and (3) serum collected within one year of diagnosis and prior to lung cancer treatment. All controls were smokers (current or former) or had a lung lesion on chest X-ray or CT suspicious for lung cancer and proven not to be cancer by either biopsy or 1 year clinical follow-up. Demographic data was collected by self-report questionnaires. Additional data for cases were acquired through clinical chart review.

Training samples were collected at 4 study centers as previously described [20]: NYU Langone Medical and Cancer Center (NYU), University of Pittsburgh Cancer Institute (PITT), Roswell Park Cancer Institute (RPCI), and Bioserve (BS). The sample cohort included cases and controls from each study center. Serum was collected from cases within 8 weeks of first biopsy-proven lung cancer diagnosis. Cases were diagnosed with pathologic or clinical stage I-IV NSCLC. The high-risk control population had a history of long-term tobacco use, including active and ex-smokers with ≥10 pack-years of cigarette smoking and those with benign pulmonary nodules. Sample selection for this study was based on our sample quality metric, described below.

The UHH validation study is a nested case/control study designed to assess the histopathological performance of the classifier. Samples were obtained from the Biobank at Thoraxklinik Heidelberg, Member of the Biomaterial Bank Heidelberg and the Biobank Platform of the German Centre for Lung Research. Approximately equal numbers of AD and SQ cases were analyzed to measure sensitivity and a small number of benign nodule controls were included to confirm specificity. The samples were collected from patients undergoing lung cancer diagnostic procedures at the Thoraxklinik; no minimum tobacco use criteria were applied.

The EDRN validation study cohort was assembled by the Lung Cancer Biomarker Group as a multicenter reference set for validating biomarkers for detection of lung cancer [47]. Samples were provided by the NCI on behalf of the EDRN. Inclusion criteria were as described above and serum collected from patients with abnormal chest X-ray or CT or at high risk for lung cancer and age ≥50 with ≥30 pack years of tobacco use. The EDRN validation study is designed to represent the population most likely to benefit from a lung cancer diagnostic test [47]. These demographics are similar to those used in the NLST study, which had a prevalence of lung cancer of 1.1% in the CT screening group [6,48].

### Ethics

All samples and clinical information were collected under Health Insurance Portability and Accountability Act (HIPAA) compliance from study participants after obtaining written informed consent under clinical research protocols approved by appropriate institutional review boards (IRBs): The University of Pittsburgh IRB (PITT); The New York University School of Medicine IRB (NYU); The Roswell Park Cancer Institute IRB (RPCI); The Cape Cod Healthcare IRB (BS), and Western IRB (UHH and EDRN).

### Sample collection procedure

Serum samples were collected following uniform processing protocols recommended by the EDRN [49]. All samples were allowed to clot and serum was recovered by centrifugation within 2–8 hours of collection and stored at -80°C. HIPAA compliant, de-identified samples were shipped frozen on dry ice to SomaLogic from the study centers. Samples were thawed twice for aliquoting (once at the site and once at SomaLogic) prior to proteomic analysis.

### Sample blinding

To prevent potential bias, a unique barcode was assigned to each sample and data record, and the key was stored in a secure database accessible only to designated study administrators. The sample blinding code was broken according to the pre-specified analysis plan. First the training set was unmasked for training the classifier. Then the biomarkers and classifier were fixed and the UHH samples were unblinded by designated study investigators. Finally, the EDRN validation set was unblinded by the Data Management and Coordinating Center of the EDRN after they received the classifier prediction results.

### Proteomic analysis

Serum samples (15 μl) were analyzed on the SOMAscan V2 proteomic assay, which measures 1033 proteins [25,50]. The SOMAscan analytes cover a broad range of proteins associated with disease physiology and biological functions, including cytokines, kinases, growth factors, proteases and their inhibitors, receptors, hormones and structural proteins [23]. SOMAscan uses novel modified DNA aptamers called SOMAmers to specifically bind protein targets in biologic samples [25,26]. All sample analyses were conducted in the Good Laboratory Practice (GLP) compliant lab at SomaLogic by trained staff as described [51]. Serum samples were distributed randomly in 96-well microtiter plates and the assay operators were blinded to case/control identity of all samples. Data for the training and two validation studies were generated in separate assay runs. Serum samples from the EDRN study were assayed with a panel assay format [51] consisting of 81 SOMAmers including the 15 candidates identified in training. A subset of the training samples was re-assayed in this format to adjust model coefficients prior to unblinding. The training and validation assay data are provided as Additional file 2 (UHH) and Additional file 3 (EDRN). Assay results are reported in Relative Fluorescence Units (RFU). Data processing was as described by Gold *et al.*[25]. Briefly, microarray images were captured and processed with a microarray scanner and associated software. Each sample in a study was normalized by aligning the median of each sample to a common reference. Inter-plate and inter-run calibration was done by applying a multiplicative scaling coefficient to each SOMAmer. These scaling factors were calculated using the eight reference calibrators on each plate.

### Sample quality assessment

Based on clinical and experimental observations, we have developed a series of protein panels and quality scores called SMVs to characterize potential preanalytic variability imparted by blood sample collection and processing [22-24]. We reported preanalytic case/control bias and site-to-site bias in a comparison of the four clinical sites in our initial lung cancer study [20] and proteins that changed with sample processing parameters such as storage and clotting time, centrifugation force, and temperature [22-24]. Three SMVs were developed from these studies that typify blood components affected by sample handling: complement (4 proteins, primarily C3 and its proteolytic fragments), cell contamination (30 intracellular leukocyte proteins released through cell lysis), and platelet contamination (16 proteins released through activation or lysis) (Additional file 4). All three SMVs can be applied to plasma, but only the complement and cell contamination SMVs are applicable to serum since platelets are activated in the clotting process. SMVs are used in two ways: (1) to assess the quality of sample collection and quality differences between study sites or cases and controls within a site and (2) to select robust biomarkers that are insensitive to preanalytic variation. Biomarkers that correlate with the SMVs should not be selected unless strict sample processing parameters can be employed uniformly. The complement and cell contamination SMVs were applied to samples from the 4 training sites to select the cohort for biomarker discovery and classifier training. Cutoffs were assigned empirically based on experimental data [22,23]. Sample quality was assessed for the validation studies, but no samples were excluded from analysis based on low quality.

### Candidate biomarker selection and classifier training

Based on the composite SMV score, 80% of the training samples were categorized as high quality and 20% as low quality but still within the sample processing protocol requirements. The rationale for selecting robust, disease specific biomarkers was to identify biomarker candidates in uniformly well collected samples that had little evidence of preanalytic variation (the 80% high quality) but ensure that these biomarkers performed consistently in lower quality samples. We have observed that classifiers trained within a sample set culled using SMVs generalize to samples outside the set, however performance degradation is sometimes observed as the sample quality degrades.

We performed non-parametric Kolmogorov-Smirnov (KS) tests to identify 15 differentially expressed analytes (Additional file 1) [19,29]. A RF classifier was built from the panel of candidate biomarkers using a backward elimination procedure that utilized the gini importance measure provided by the RF classifier [52,53]. RF is a decision-tree based method and predictions are influenced by the gini importance score of each biomarker. To improve training prediction accuracy, iterative bootstrap aggregation, or bagging, combined with out of bag error estimation was used to reduce the training variance [53]. The gini importance is a measure of the effectiveness of a biomarker for correctly classifying samples in the training set and can be used to eliminate markers that are less vital to the performance of the classifier. The backward elimination procedure was initiated by building a RF classifier that included all candidate biomarkers. The least important biomarker was then eliminated and a new model was built with the remaining biomarkers. This procedure continued until only single biomarkers remained. The final 7-marker panel was selected because it provided the best balance between the highest AUC and the lowest number of markers in the model. RF case/control prediction probability scores span from 0 to 1, and for binary performance estimations we selected a case threshold of 0.45 or greater. The final classifier was then applied to make blinded predictions on the two independent validation sets.

The study design and execution were conducted according to accepted best practices [54]. Analyses were performed with R statistical software version 2.10.1. Functional analysis was performed with DAVID Bioinformatics Resources version 6.7 [55].

## Abbreviations

AD: Adenocarcinoma; AUC: Area under the curve; NPV: Negative predictive value; NSCLC: Non-small cell lung cancer; PPV: Positive predictive value; RFU: Relative fluorescent units; RF: Random forest; ROC: Receiver operating characteristic; SMV: Sample mapping vector; SQ: Squamous cell carcinoma.

## Competing interests

ENB, MRM, RMO and SAW are employed by SomaLogic, Inc. and are listed as inventors on SomaLogic lung cancer biomarker patent applications. The other authors have nothing to declare.

## Authors’ contributions

MRM, RMO, ENB and SAW designed the study. JMS, WLB, JLW, DOW, HIP, WNR, TM, and MM provided clinical samples and participated in the design of the studies. WF and YEM participated in the histological analysis. MRM and RMO performed statistical analysis. RMO wrote the manuscript with input from all others. All authors evaluated and interpreted the analyzed data, and critically reviewed and approved the manuscript.

## Supplementary Material

Additional file 1 Candidate protein biomarkers identified in training. Click here for file

Additional file 2 **UHH Validation, attached as separate csv file.** Training and validation data for the UHH validation study. Click here for file

Additional file 3 **EDRN Validation, attached as separate csv file.** Training and validation data for the EDRN validation study. A subset of the original training samples was repeated in this study to calibrate the model for the panel format, and this data is included in Additional file 3. Click here for file

Additional file 4 Protein components of the SMV vectors. Click here for file
