# Supplementary material for: Validation of a blood protein signature for non-small cell lung cancer
Source: Clin Proteomics. 2014 Aug 1;11(1):32. doi: 10.1186/1559-0275-11-32 (PMC4123246; doi:10.1186/1559-0275-11-32)
Supplement: Additional file 1 — Candidate protein biomarkers identified in training. [file 1559-0275-11-32-S1.doc]

**Additional File 1**: Candidate protein biomarkers identified in training

| **Protein** | **Gene ID** | **SwissProt ID** |
| --- | --- | --- |
| BMP1 | 649 | P13497 |
| C9 | 735 | P02748 |
| CA6 | 765 | P23280 |
| CHRDL1 | 91851 | Q9BU40 |
| CNDP1 | 84735 | Q96KN2 |
| CRP | 1401 | P02741 |
| EGFR | 1956 | P00533 |
| IGFBP2 | 3485 | P18065 |
| KIT | 3815 | P10721 |
| KLK7 | 5650 | P49862 |
| LRIG3 | 121227 | Q6UXM1 |
| MMP12 | 4321 | P39900 |
| MMP7 | 4316 | P09237 |
| MRC1 | 4360 | P22897 |
| SERPINA3 | 12 | P01011 |
