# Supplementary material for: Validation of a blood protein signature for non-small cell lung cancer
Source: Clin Proteomics. 2014 Aug 1;11(1):32. doi: 10.1186/1559-0275-11-32 (PMC4123246; doi:10.1186/1559-0275-11-32)
Supplement: Additional file 4 — Protein components of the SMV vectors. [file 1559-0275-11-32-S4.doc]

**Additional File 2**: Protein components of the SMV vectors

| **#** | **Protein** | **Gene ID** | **Swiss Prot ID** | **Protein Name** | **Cell Contamination** | **Platelet Contamination** | **Complement** |
| --- | --- | --- | --- | --- | --- | --- | --- |
| 1 | ACP1 | 52 | P24666 | PPAC | X |  |  |
| 2 | ADRBK1 | 156 | P25098 | BARK1 | X |  |  |
| 3 | CAPN1-CAPNS1 | 823; 826 | P07384; P04632 | Calpain I | X |  |  |
| 4 | CASP3 | 836 | P42574 | Caspase-3 | X |  |  |
| 5 | CSK | 1445 | P41240 | CSK | X |  |  |
| 6 | EIF5A | 1984 | P63241 | eIF-5A-1 | X |  |  |
| 7 | GDI2 | 2665 | P50395 | Rab GDP dissociation inhibitor beta | X |  |  |
| 8 | GSK3A | 2931 | P49840 | GSK-3 alpha | X |  |  |
| 9 | HSP90AA1 | 3320 | P07900 | HSP 90alpha | X |  |  |
| 10 | HSP90AB1 | 3326 | P08238 | HSP 90beta | X |  |  |
| 11 | HSPA1A | 3303 | P08107 | HSP 70 | X |  |  |
| 12 | IDE | 3416 | P14735 | Insulysin | X |  |  |
| 13 | KPNB1 | 3837 | Q14974 | Importin beta1 | X |  |  |
| 14 | MAPK1 | 5594 | P28482 | MAPK1 | X |  |  |
| 15 | MAPK3 | 5595 | P27361 | MAPK3 | X |  |  |
| 16 | MAPKAPK3 | 7867 | Q16644 | MAPKAPK3 | X |  |  |
| 17 | MDH1 | 4190 | P40925 | MDHC | X |  |  |
| 18 | NACA | 4666 | Q13765 | NACalpha | X |  |  |
| 19 | PAFAH1B2 | 5049 | P68402 | PAFAH beta subunit | X |  |  |
| 20 | PPIA | 5478 | P62937 | Cyclophilin A | X |  |  |
| 21 | PRDX1 | 5052 | Q06830 | Peroxiredoxin-1 | X |  |  |
| 22 | PRKACA | 5566 | P17612 | PRKA C-alpha | X |  |  |
| 23 | PRKCI | 5584 | P41743 | PRKCI | X |  |  |
| 24 | RAC1 | 5879 | P63000 | RAC1 | X |  |  |
| 25 | RPS6KA3 | 6197 | P51812 | RPS6Kalpha3 | X |  |  |
| 26 | RPS7 | 6201 | P62081 | RS7 | X |  |  |
| 27 | STIP1 | 10963 | P31948 | Stress-induced-phosphoprotein 1 | X |  |  |
| 28 | UBE2I | 7329 | P63279 | UBC9 | X |  |  |
| 29 | UBE2N | 7334 | P61088 | UBE2N | X |  |  |
| 30 | UFC1 | 51506 | Q9Y3C8 | UFC1 | X |  |  |
| 31 | ANGPT1 | 284 | Q15389 | Angiopoietin-1 |  | X |  |
| 32 | APP | 351 | P05067 | amyloid precursor protein |  | X |  |
| 33 | BDNF | 627 | P23560 | BDNF |  | X |  |
| 34 | CCL5 | 6352 | P13501 | RANTES |  | X |  |
| 35 | CTSA | 5476 | P10619 | Cathepsin A |  | X |  |
| 36 | DKK4 | 27121 | Q9UBT3 | Dkk-4 |  | X |  |
| 37 | MDK | 4192 | P21741 | Midkine |  | X |  |
| 38 | MMP9 | 4318 | P14780 | MMP-9 |  | X |  |
| 39 | PDGFB | 5155 | P01127 | PDGF-BB |  | X |  |
| 40 | PF4 | 5196 | P02776 | PF-4 |  | X |  |
| 41 | PPBP | 5473 | P02775 | NAP-2 |  | X |  |
| 42 | SERPINE1 | 5054 | P05121 | PAI-1 |  | X |  |
| 43 | SERPINE2 | 5270 | P07093 | Protease nexin I |  | X |  |
| 44 | SPARC | 6678 | P09486 | Osteonectin |  | X |  |
| 45 | THBS1 | 7057 | P07996 | Thrombospondin-1 |  | X |  |
| 46 | TIMP3 | 7078 | P35625 | TIMP-3 |  | X |  |
| 47 | C3 | 718 | P01024 | iC3b |  |  | X |
| 48 | C3 | 718 | P01024 | C3 |  |  | X |
| 49 | C3 | 718 | P01024 | C3adesArg |  |  | X |
| 50 | LTA4H | 4048 | P09960 | LTA-4 hydrolase |  |  | X |
